# National variability in Americans’ COVID-19 protective behaviors: Implications for vaccine roll-out

**DOI:** 10.1371/journal.pone.0259257

**Published:** 2021-11-05

**Authors:** John A. Schneider, Bruce G. Taylor, Anna L. Hotton, Phoebe A. Lamuda, Jonathan Ozik, Qinyun Lin, Elizabeth Flanagan, Mai Tuyet Pho, Marynia Kolak, Russell Brewer, Jade Pagkas-Bather, Harold A. Pollack

**Affiliations:** 1 Department of Medicine, University of Chicago, Chicago, Illinois, United States of America; 2 NORC, Chicago, Illinois, United States of America; 3 Argonne National Laboratory, Chicago, Illinois, United States of America; 4 Center for Spatial Data Science, University of Chicago, Chicago, Illinois, United States of America; 5 Crown Family School of Social Work, Policy, and Practice, University of Chicago, Chicago, Illinois, United States of America; Kyushu Daigaku, JAPAN

## Abstract

Protective behaviors such as mask wearing and physical distancing are critical to slow the spread of COVID-19, even in the context of vaccine scale-up. Understanding the variation in self-reported COVID-19 protective behaviors is critical to developing public health messaging. The purpose of the study is to provide nationally representative estimates of five self-reported COVID-19 protective behaviors and correlates of such behaviors. In this cross-sectional survey study of US adults, surveys were administered via internet and telephone. Adults were surveyed from April 30-May 4, 2020, a time of peaking COVID-19 incidence within the US. Participants were recruited from the probability-based AmeriSpeak® national panel. Brief surveys were completed by 994 adults, with 73.0% of respondents reported mask wearing, 82.7% reported physical distancing, 75.1% reported crowd avoidance, 89.8% reported increased hand-washing, and 7.7% reported having prior COVID-19 testing. Multivariate analysis (p critical value .05) indicates that women were more likely to report protective behaviors than men, as were those over age 60. Respondents who self-identified as having low incomes, histories of criminal justice involvement, and Republican Party affiliation, were less likely to report four protective behaviors, though Republicans and individuals with criminal justice histories were more likely to report having received COVID-19 testing. The majority of Americans engaged in COVID-19 protective behaviors, with low-income Americans, those with histories of criminal justice involvement, and self-identified Republicans less likely to engage in these preventive behaviors. Culturally competent public health messaging and interventions might focus on these latter groups to prevent future infections. These findings will remain highly relevant even with vaccines widely available, given the complementarities between vaccines and protective behaviors, as well as the many challenges in delivering vaccines.

## Introduction

The United States is currently experiencing the largest COVID-19 epidemic in the world with 20% of the world’s cases and the most COVID-19 deaths [1]. Clear disparities in COVID-19 infection and death rates exist within the US, with African Americans and Hispanic populations experiencing greater disease burden than other racial/ethnic groups [2]. COVID-19 data from several countries reflects a higher case fatality rate for elderly populations [3]. Evidence suggests an association between COVID-19 rates and lower income likely associated with multiple factors including low income, criminal justice involvement (CJI), substance use disorders (SUD), particularly opioid use disorder (OUD), overpopulated and poor housing conditions, dwelling in multigenerational homes, use of public transport, and higher rates of comorbidities [4–8].

Various public health agencies including the WHO [9] and Centers for Disease Control and Prevention [10] have issued guidance on proven COVID-19 protective behaviors to complement vaccine implementation [11]. Recommended COVID-19 protective behaviors include physical distancing, use of face coverings, avoidance of large gatherings, and basic hand hygiene measures, among other measures to prevent transmission such as COVID-19 testing [9, 12].

Despite these calls for COVID-19 protective behaviors, little is known about uptake of these practices, and how these behaviors vary by demographic and other individual and community characteristics [13]. It is unclear, for example, whether a growing recognition of inequities in COVID-19 transmission and mortality has translated into effective protective behaviors, particularly within populations facing the greatest risks. Such information is critical to identifying gaps in prevention, and may help to identify individuals and communities for further engagement and focused public health messaging.

This analysis explores these questions by investigating protective behaviors in a national sample of the US adult population. We use a national probability-based survey linked to aggregate level data on COVID-19 hotspots and state adopted non-pharmaceutical interventions (NPIs) related to COVID-19. In particular, we explore prevalence of COVID-19 protective behaviors and individual and contextual-level correlates of protective behaviors and NPIs across the US, particularly among subgroups of greatest vulnerability.

## Methods

Survey data were collected from a cross-sectional sample of 994 US adults drawn from AmeriSpeak®, a probability-based non-institutionalized panel of over 45,000 panel members designed to be representative of the US household population. Randomly selected households were sampled using area probability and address-based sampling, with known selection probabilities from the NORC National Sample Frame [14]. Sampled households are then contacted by US mail, telephone, and face-to-face interviewers to capture hard-to-reach respondents. For this paper, our sample of 994 participants provided good statistical precision. With a design effect of 1.8, based on a margin of error for a 50% statistic (the most conservative case) at 95% confidence level our statistical precision is +/- 4.2%.

The panel provides sample coverage of approximately 97% of the US household population. Prior methodology comparisons indicate that the resulting AmeriSpeak sample broadly replicates the US Census American Community Survey sample on key demographic measures, including sex, age, race/ethnicity, education, marital status, employment, income, region, and home Internet access [15, 16]. The annual panel retention rate is 85% [17]. AmeriSpeak panel’s weighted household recruitment rate, which includes a second stage of recruitment for initial non-responders to capture harder-to-reach populations, is 37%, one of the highest for comparable panels [16]. This study was carried out in accordance with the approval that the research team received from the NORC at the University of Chicago Institutional Review Board (IRB00000967), under its Federal-wide Assurance (FWA00000142). Voluntary and informed consent was obtained from all participants either by the participant checking a time-stamped box online for those completing the web version of the survey or consenting verbally for those completing a survey by phone (with a NORC interviewer checking a box on the survey form for the participant and initialing and dating when consent was received).

### Measures

#### Dependent variables

*Five key COVID-19 protective behaviors*. Respondents were queried about their COVID-19 protective behaviors to include: “Wearing a mask when leaving home,” “Keeping a 6-foot radius when interacting with people you do not live with,” “Limiting interactions with others to groups of 10 or less” and “Washed/sanitized hands more than usual.” Respondent were also asked if they had been tested (no/yes) for COVID-19. COVID-19 related items were taken from NIH’s PhenX Toolkit COVID-19 Protocols, specifically from the work of Cawthon and colleagues [18].

#### Independent variables

*Respondent-level background variables*. Age was collected as a continuous variable and categorized as 18–30, 31–59, and 60 and older. We measured biological sex (Female = 0 and Male = 1), race/ethnicity (non-Hispanic White was used as the reference category, hereafter White), non-Hispanic Black (Black), non-Hispanic Other/Mixed (Other/Mixed), non-Hispanic Asian (Asian), Hispanic, years of education (No college at all = 0, at least some college = 1), employment (not employed = 0 and employed = 1), income (below state median income = 0 and above state median income = 1), personal lifetime history of CJI (yes/no to a self-reported conviction for a misdemeanor or felony crime or incarceration in jail or prison), personal lifetime history of non-medical opioid use (yes/no to use of opioids or prescription pain medication illicitly obtained or used in a way not prescribed by a doctor) and self-reported political party affiliation (Democrat, lean Democrat, don’t lean/independent party or other, lean Republican, Republican).

*Local contextual variables*. Based on the respondent’s home address we measured the US Census region of the respondent’s home (South [referent], Northeast, Midwest, and West). Next, we included a county-level dichotomous COVID-19 hotspot variable based on whether the county was identified as an emerging statistical hotspot for all days during the survey period. Specifically, we implemented Local Indicators of Spatial Association (LISA) analyses [19–21] on population adjusted 7-day average new confirmed COVID-19 cases, to detect clusters of contiguous counties that were statistically similar in having high 7-day average new COVID-19 case rates. We used a conditional permutation approach for inference, with 999 Monte Carlo permutations and a pseudo p-value threshold of 0.05. A hotspot cluster thus corresponds to counties that had high rates, and were surrounded by counties with high rates, relative to the rest of the country (Exhibit 4). We used USAFacts [22] data for COVID-19 case rates, and GeoDa statistical software for LISA calculation [23].

We included a state policy count measure indicating whether the state implemented one or more NPI policies (i.e., requirements that residents stay home, ban on gatherings, requirement that residents wear face coverings, restrictions on restaurants, requirement that businesses close.)

#### Analytic plan

Multivariable logistic regression models were conducted for each outcome (face covering, social distancing, hand hygiene, limited gatherings, and COVID-19 testing). Variables were selected for inclusion in the multivariable models based on *a priori* hypotheses about their relationships with COVID-19 protective behaviors and all variables were retained regardless of statistical significance. All analyses (p critical value set at .05) used data weighted to national census benchmarks, taking into account selection probabilities (balanced by sex, age, education, race/ethnicity, and region)[17] and non-response (using a response propensity approach calculating the conditional probability that a particular respondent completed the survey given observed covariates) [24]. Missing data were handled with listwise deletion. Analyses were conducted using IBM SPSS Statistics Software Version 27.0.1.

## Results

### Sample description

The survey was completed by 994 of those invited from the larger AmeriSpeak panel (24%) with 17 respondents (2%) excluded from the analysis due to missing data, producing a final sample of 977 respondents. AmeriSpeak has been designed to address response rate and sampling issues through sample weights and non-response adjustments [16, 25–27]. Participants (498 female and 479 male) aged 18–89 were included in all analyses. Sociodemographic characteristics are summarized in Table 1. Thirty-eight percent of respondents lived in the South, 18% in the Northeast, 20% in the Midwest, and 24% in the West. About 11% of the sample (11.4%) experienced CJI and 7% had a personal experience with opioid misuse. On average, participants’ states of residence had about five (5.2) COVID-19-related safeguard practices in place at the time of the survey.

**Table 1:** Characteristics of adult respondents, April 30-May 4, 2020 (n = 977).

	%	(95% CI)
**Age**		
18–30	22.0%	(19.4, 24.6)
31–59	48.3%	(45.1, 51.4)
60+	29.8%	(26.9, 32.7)
**Male**	49.0%	(45.8, 52.1)
**Race/ethnicity** ^a^		
White	62.8%	(59.7, 65.98)
Hispanic	16.9%	(14.6, 19.3)
Black	11.8%	(9.8, 13.9)
Asian, non-Hispanic	3.5%	(2.3, 4.7)
Other/Two or more	5.0%	(3.6, 6.4)
**Census region respondent resides**		
Northeast	17.6%	(15.2, 20.0)
Midwest	20.6%	(18.1, 23.2)
South	37.8%	(34.8, 40.8)
West	24.0%	(21.3, 26.7)
**Educational attainment**		
< HS graduate	9.9%	(8.0, 11.8)
HS graduate or equivalent	27.7%	(24.8, 30.5)
Some college	27.8%	(24.9, 30.6)
BA or above	34.7%	(31.7, 37.7)
**Employment Status**		
Not working–on temporary layoff from a job	1.0%	(0.4, 1.7)
Not working–looking for work	9.4%	(7.6, 11.2)
Not working–retired	17.6%	(15.2, 20.0)
Not working–disabled	6.9%	(5.3, 8.4)
Not working–other	10.0%	(8.1, 11.9)
Working–as a paid employee	47.4%	(44.3, 50.5)
Working–self-employed	7.7%	(6.0, 9.4)
**Household Income**		
<$25,000	21.4%	(18.8, 23.9)
$25,000-$49,999	25.1%	(22.4, 27.8)
$50,000-$84,999	23.5%	(20.8, 26.1)
$85,000-$149,999	21.5%	(18.9, 24.0)
$150,000+	8.5%	(6.9, 10.4)
**Opioid Misuse and Criminal Justice History**		
Opioid use^b^ (% yes)	7.0%	(5.4, 8.6)
Criminal justice involvement^c^ (% yes)	11.4%	(9.4, 13.3)
**COVID-19 Cases**		
Presence of a COVID Hotspot, Adjusted^d^	10.2%	(8.3, 12.1)
**Prevention Policies**		
State Policy Counts^e^ (Mean, SD)	5.15 (0.77)	(5.1, 5.2)
**COVID-19 testing**		
Tested	7.7%	(6.0, 9.3)
Positive (of those tested)	10.0%	(3.1,16.8)
**Political Party Affiliation**		
Democrat	33.9%	(30.9, 36.9)
Lean Democrat	10.6%	(8.7, 12.5)
Neither/Don’t Lean/Independent	18.3%	(15.9, 20.8)
Lean Republican	11.3%	(9.3, 13.3)
Republican	25.9%	(23.1, 28.6)

^a^ Race categories are mutually exclusive.

^b^ Opioid misuse is defined in the survey as ever used opioids illicitly obtained or used in a way not prescribed by a doctor.

^c^ Criminal justice involvement is defined as convicted of any misdemeanor or felony crime and/or incarcerated in jail or prison.

^d^ Whether the county identified as a hotspot during the survey period. A hotspot was defined as having a high number of daily cases in the county and surrounded by counties with a high number of cases based on daily new cases adjusted for population size.

^e^ Number of state policies related to COVID implemented prior to April 30, 2020 period.

#### COVID-19 protective behaviors

COVID-19 testing was infrequent at the time of the survey (April 30 and May 4, 2020); only 7.7% of respondents reported having been tested and of those a 10.0% positivity rate.

**Fig 1:**
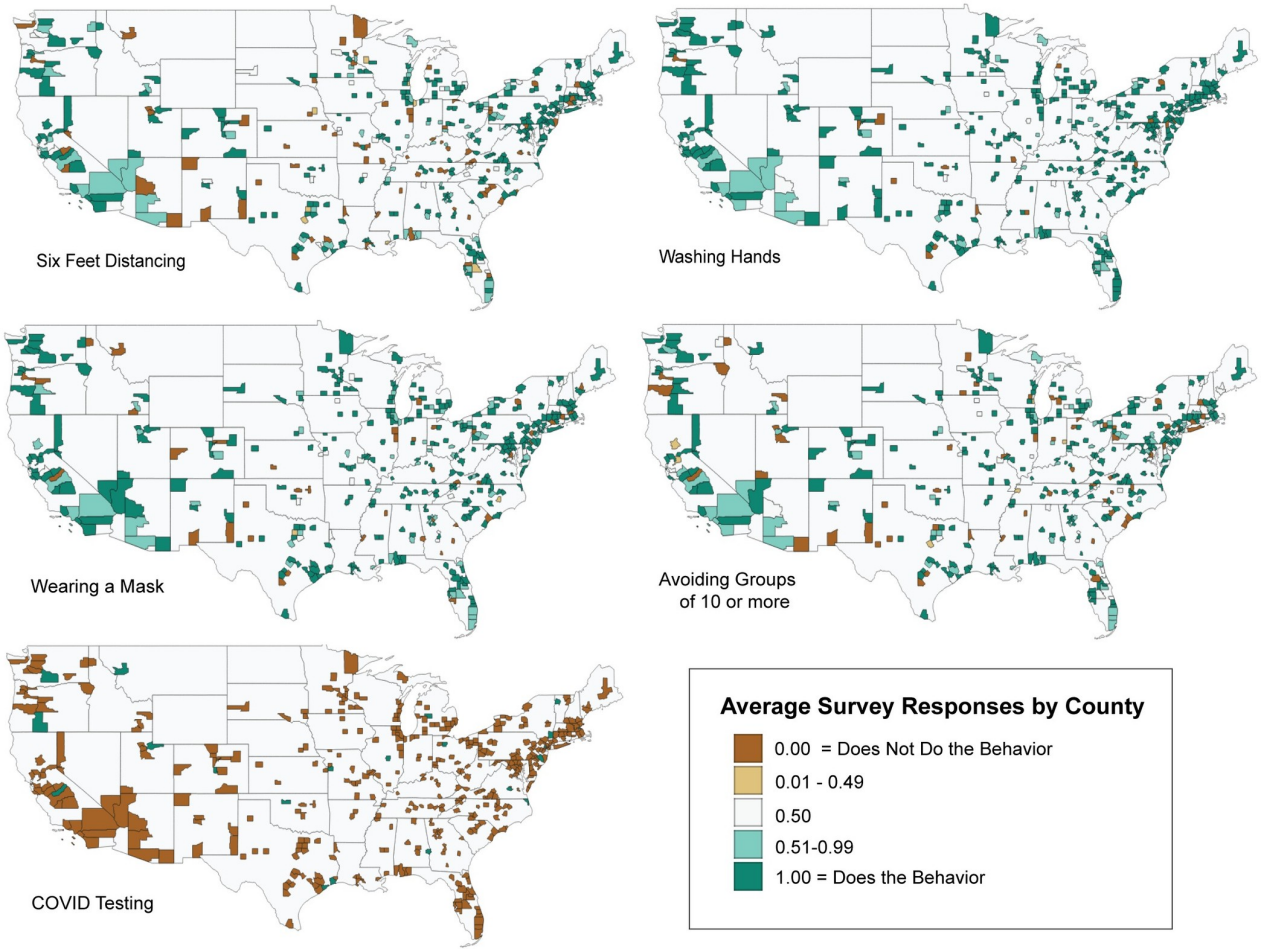
Average survey response by county for five COVID-19 prevention acts. Only counties with survey respondents in the continental US are shown. While survey responses were binary (Agree = 1, Does not agree = 0), results are averaged at the county-scale.

As seen in Fig 1, average survey responses by county were mapped across all protective behaviors reviewed within the continental United States. The lack of COVID-19 testing is clear, in contrast with multiple other measures more often taken by survey respondents. Most counties indicate hand-washing as a key measure used, regardless of location, when viewed in aggregate. Six-feet social distancing, avoiding social contact, avoiding groups of 10 or more, and wearing a mask all have spatially heterogeneous patterns at the county-scale that vary by location.

Almost all respondents reported taking at least one protective behavior to reduce COVID-19 risk. As seen in Table 2, 73% reported that they wore face coverings when out in public; 83% reported that they maintained distancing of six feet or greater; 75% reported that they stayed in groups of ten or less. About 90% of the respondents reported that they washed hands more than usual. While 54% report using all four prevention measures to reduce COVID-19 risk (Table 2), more than two-thirds of the respondents who report one of the measures tended to report a second prevention measure. For example, more than two-thirds of those who reported using face coverings also reported using physical distancing (67%), and good hand hygiene (70%) and those who reported using physical distancing also reported staying in groups of 10 or less (68%) and good hand hygiene (77%).

**Table 2:** Overlap of protective practices among respondents.

Protective Practice (No. (%))					
	Face Covering	Social Distance	Groups 10 or Less	Hand Hygiene	
	731 (73.1)	828 (82.7)	753 (75.1)	899 (89.8)	
**Face Covering**		670 (66.9)	598 (59.6)	697 (69.5)	
**Social Distance**			680 (67.8)	770 (76.9)	
**Groups 10 Or Less**				702 (70.0)	
	**Hand Hygiene**	641 (63.9)	575 (57.4)		**Face Covering**
	**Face covering**		566 (56.5)		**Social Distance**
	**Social Distance**			642 (64.0)	**Groups 10 or Less**
**Face Covering + Social Distance + Groups 10 or Less + Hand Hygiene**			543 (54.2)		

The number and percent of respondents self-reporting one protective practice is shaded light blue, two protective behaviors is shaded blue, and three protective behaviors is shaded grey. The number and percent of respondents self-reporting all four protective behaviors is shaded orange.

As seen in Table 3, controlling for other factors, face covering was markedly more common among older respondents 60 and over compared to younger respondents 18–30 years old (adjusted odds ratio (AOR) = 2.55, 95% CI, 1.53–4.24), and was significantly less likely among men (AOR = 0.59, 95% CI, 0.43–0.83). When compared to Whites, reporting a Hispanic identity (AOR = 1.68, 95% CI, 1.03–2.74) or affiliating with the Democratic Party (AOR = 3.15, 95% CI, 2.04–4.87) was independently associated with face coverings. Those with CJI histories were less likely (AOR = 0.42, 95% CI, 0.26–0.68) to report use of face coverings.

**Table 3:** Multivariable logistic regression for COVID-19 preventive behaviors.

	Wear Mask when Leaving Home	Maintain Six Feet of Distance from Others	Limit Interactions to Groups of 10 or Less	Wash Hands More Often than Usual	Respondent Tested for COVID-19
	(n = 963)	(n = 963)	(n = 962)	(n = 963)	(n = 953)
	AOR (95% CI)	AOR (95% CI)	AOR (95% CI)	AOR (95% CI)	AOR (95% CI)
Age					
18–30 (reference)					
31–59	0.79 (0.52, 1.20)	2.70 (1.70, 4.30)***	0.86 (0.56, 1.30)	0.92 (0.51, 1.67)	1.40 (0.72, 2.73)
60+	2.55 (1.53, 4.24)***	4.80 (2.68, 8.62)***	1.20 (0.73, 1.96)	2.05 (0.96, 4.36)	0.68 (0.28, 1.65)
Sex					
Male	0.60 (0.43, 0.83)**	0.91 (0.61, 1.35)	0.62 (0.44, 0.85)**	1.14 (0.69, 1.86)	0.69 (0.40, 1.19)
Race^a^					
White (reference)					
Black	0.92 (0.55, 1.54)	0.37 (0.21, 0.67)**	0.41 (0.25, 0.68)***	0.55 (0.27, 1.10)	3.00 (1.42, 6.32)**
Other/Mixed	1.87 (0.85, 4.11)	1.20 (0.49,2.95)	1.26 (0.56, 2.81)	0.24 (0.11, 0.53)***	2.41 (0.84, 6.88)
Asian	1.14 (0.47, 2.75)	0.84 (0.29, 2.44)	1.30 (0.49, 3.48)	0.81 (0.19, 3.41)	3.94 (1.26, 12.37)*
Hispanic	1.68 (1.02, 2.74)*	0.97 (0.55, 1.71)	0.45 (0.29, 0.70)***	2.48 (1.00, 6.18)	1.91 (0.86, 4.22)
Education					
Some college or above	1.10 (0.78, 1.55)	0.87 (0.58, 1.31)	1.17 (0.83, 1.65)	2.59(1.56, 4.32)***	0.62 (0.35, 1.07)
Income					
Below state median income	0.49 (0.34, 0.70)***	0.52 (0.34, 0.79)**	0.57 (0.40, 0.81)**	0.63(0.37, 1.08)	1.98 (1.09, 3.61)*
Employment					
Employed	1.52 (1.07, 2.16)*	1.24 (0.82, 1.86)	1.16 (0.82, 1.54)	1.47 (0.88, 2.46)	1.15 (0.66, 2.02)
Opioid Misuse History^b^					
Yes	1.28 (0.67, 2.43)	2.39 (1.03, 5.54)*	0.75 (0.41, 1.39)	0.71 (0.30, 1.69)	1.11 (0.46, 2.68)
Criminal Justice History^c^					
Yes	0.42 (0.26, 0.68)***	0.31 (0.18, 0.54)***	1.04 (0.63, 1.71)	0.45 (0.24, 0.84)*	6.09 (3.24, 11.45)***
State Policy Count^d^	1.11 (0.97, 1.25)	0.95 (0.81, 1.12)	1.08 (0.94, 1.23)	1.07 (0.90, 1.28)	1.11 (0.88, 1.39)
US Region					
South (reference)					
Northeast	2.42 (1.30, 4.50)**	1.67 (0.80, 3.48)	0.80 (0.45, 1.42)	1.53 (0.56, 4.21)	0.83 (0.31, 2.24)
Midwest	0.98 (0.65, 1.49)	1.32 (0.78, 2.22)	1.06 (0.68, 1.67)	0.60 (0.33, 1.09)	1.12 (0.54, 2.32)
West	0.92 (0.60, 1.43)	1.11 (0.65, 1.89)	0.73 (0.47, 1.12)	0.63 (0.32, 1.25)	1.31 (0.62, 2.76)
Emerging COVID-19 Hotspot^e^					
Yes	0.64 (0.32, 1.28)	0.75 (0.33, 1.71)	1.36 (0.68, 2.69)	0.71 (0.24, 2.11)	1.34 (0.45, 3.98)
Political Affiliation					
Republican (reference)					
Democrat	3.15 (2.04, 4.87)***	7.19 (4.02, 12.88)***	2.19 (1.39, 3.43)**	3.40 (1.65, 6.98)**	0.37 (0.18, 0.77)**
Lean Democrat	3.12 (1.68, 5.81)***	6.04 (2.72, 13.40)***	1.31 (0.72, 2.37)	2.04 (0.81, 5.13)	0.25 (0.08, 0.84)*
Don’t Lean/ Independent	1.46 (0.92, 2.33)	2.18 (1.27, 3.72)**	0.76(0.47, 1.21)	1.17 (0.61, 2.24)	0.50 (0.23, 1.08)
Lean Republican	1.33 (0.79, 2.26)	1.56 (0.85, 2.86)	1.02 (0.59, 1.76)	1.17 (0.55, 2.50)	0.71 (0.30, 1.65)
Nagelkerke’s R^2^	0.19	0.24	0.135	0.19	0.19

Abbreviations: AOR, adjusted odds ratio; CI, confidence interval.

*p < .05

**p < .01

***p < .001.

^a^ Race categories were mutually exclusive.

^b^ Opioid misuse is defined in the survey as ever used opioids/prescription pain medication illicitly obtained or used in a way not prescribed by a doctor.

^c^ Criminal justice involvement is defined in the survey as convicted of any misdemeanor or felony crime and/or incarcerated in jail or prison.

^d^ Number of state policies related to COVID-19 implemented prior to April 30, 2020 period.

^e^ Whether the county identified as a hotspot all days during the survey period. A hotspot was defined as having a high number of daily cases in the county and surrounded by counties with a high number of cases based on daily new cases for every day, adjusted for population size.

Social distancing was more common among older respondents 31–59 (AOR = 2.70, 95% CI, 1.69–4.30), and those age 60 and over (AOR = 4.80, 95% CI, 2.68–8.62) compared to younger respondents 18–30 years old. In addition, Black race (AOR = 0.37, 95% CI, 0.21–0.67), incomes below their state median (AOR = 0.52, 95% CI, 0.34–0.79), CJI history (AOR = 0.31, 95% CI, 0.18–0.54), non-medical opioid use (AOR = 2.39, 95% CI, 1.03–5.54) and affiliation with the Democratic Party (AOR = 7.19, 95% CI, 4.02–12.88), were all associated with social distancing.

COVID-19 testing was significantly independently associated with Black race (AOR = 3.00, 95% CI, 1.42–6.32), Asian race (AOR = 3.94, 95% CI, 1.26–12.37), incomes below their state median (AOR = 1.98, 95% CI, 1.09–3.61), individuals with justice involvement histories (AOR = 6.09, 95% CI, 3.24–11.45) and Democratic Party affiliation (AOR = 0.37, 95% CI, 0.18–0.77).

## Discussion

US adults report high uptake of at least some COVID-19 protective behaviors, though uptake of multiple measures at once was more modest. Respondents reported low overall rates of COVID-19 testing. This finding was expected, given the strong geographic clustering of COVID-19 cases at this phase of the pandemic and the limited availability of testing nationwide.

Demographic characteristics, economic and social circumstance, and political values are all associated with specific protective behaviors. Older respondents and respondents affiliating with the Democratic Party reported higher uptake of such practices (similar to a convenience sample study done early in the pandemic in the US that examined political affiliation)[13]; while more marginalized populations such as individuals with CJI histories and those with lower income reported fewer protective behaviors. Those with prior CJI histories and lower incomes might have lower reported use of protective behaviors because of the nature of their employment. These may also reflect less access to prevention resources. These patterns may also reflect broader vulnerabilities such as crowded housing or vulnerability to homelessness.

CJI populations and respondents with Republican political affiliation were more likely to have been COVID-19 tested. Individuals with CJI experience may have been more likely to be tested due to involuntary confinement with others, congregate living in jails and prisons or due to contact with community-based system that required such testing. Republican political affiliation may have used COVID-19 testing as an alternative to other preventive behaviors such as the use of face coverings, which are less acceptable among this sub-population.

Diverse demographic factors, including age and gender, have been found to affect the general adoption of public health preventive practices [28, 29]. In a Kaiser study, men were less likely to report taking prevention actions [30]. A study of Turkish adults found that younger people, females, and those with higher education reported more preventive behaviors [31].

This study’s US sample displayed a slightly different pattern. Older respondents reported higher rates of protective behaviors, perhaps reflecting early and clear public awareness of the sharp age gradients in COVID-19 morbidity and mortality. We found no consistent relationship between education and protective behaviors. However, we found that females were more likely to use facial coverings and restrict themselves to groups of 10 or less.

Individuals with OUD are particularly vulnerable to COVID-19 infection and worse outcomes from the disease, including hospitalization and death [7]. We did not find a clear relationship between opioid use and COVID-19 protective behaviors (other than physical distancing) despite clear relationships between OUD and accessing healthcare, restrictions on substitution therapy during COVID-19, increased risk of life-threatening withdrawals, and limitations on obtaining support due to the shuttering of treatment centers [32].

A model to help with building on our results in future research are socio-physical models. Socio-physical models have been used to interpret the interactions of complex social systems such as public opinions, behavior of crowds and collective decision making [33]. Socio-physical models suggest that the COVID-19 protective behaviors examined in our study may be shaped, at least in part, by social influence [33]. That is, individuals are likely to be influenced by others they interact with and are more likely to adopt similar behavior or attitudes as those around them [33]. This could explain some of our results such as political affiliation, with Democrats likely spending more time around other Democrats which helps reinforce their adoption of COVID-19 protective behaviors. Future research should consider using social network analysis to study smaller networks than our national study. Such research would assess if and how the COVID-19 protective behaviors documented in our study are interrelated within social networks, as documented in Litwin and Levinsky’s research with an older population [34] and as suggested by socio-physical model theory.

Building on ‘vaccination game’ research, which gives a mathematical framework to account for this interrelated behavior not only for epidemiologic dynamics but also for the voluntary mitigation risk behavior of individuals who face pandemics [35], can also be useful in future work. Kabir, Risa and Tanimoto provided an excellent recent example of using a game model, combining the mathematical models of epidemiology with evolutionary game theory, to study mask wearing during the COVID-19 pandemic [36]. Kabir and colleagues quantify how people wearing masks directly benefit the wearer and brings advantage to other people during a pandemic, based on a social learning process that accounts for the risk of infection and mask wearing intangible costs. The work of Kabir and colleagues reveals a diverse and rich social dilemma structure and sociological dimension to mask wearing. Our study did not have the data to explore this dimension but future research should consider examining this further.

Other next steps based on our research include using tailored and community-specific engagement to assist with the deployment of COVID-19 prevention interventions. This is critically important given that Black/African Americans, Hispanic/Latinx community members and other minoritized populations historically have either had limited or delayed access to prevention interventions [37, 38]. These communities may harbor longstanding mistrust in health care and government institutions which represents an additional barrier to COVID-19 vaccination [38]. Striking differences in self-reported protective practices by party identification underscore the need for culturally competent public health messaging that crosses partisan divides [39].

### Study limitations

This analysis did not allow for causal or other temporal inferences with cross-sectional data. Our data were also collected during a particular early period in the COVID-19 pandemic and patterns of protective practices may have shifted over time. While fifteen percent of our sample reported histories of either opioid misuse or CJI, our household study did not include individuals presumably at the highest risk (e.g., those currently incarcerated). We also lack measures of COVID protective behaviors and risk-factors among individuals with current or very recent histories of CJI involvement or opioid use; something that will be examined in detail through the NIH’s RADx-UP initiative [40]. We also did not have a more detailed measure of political affiliation capturing more extreme ideologies (e.g., "far right", "far left"), and therefore might have failed to capture more extreme behaviors regarding adoption of COVID-19 prevention guidance.

As with other household surveys, this study displayed a modest response rate. Nevertheless, the AmeriSpeak panel’s response rate of 37% is one of the highest for comparable national probability-based household panels [16]. We weighted the data to national census benchmarks, taking into account selection probabilities and addressed possible non-response bias with statistical weights and non-response adjustments.

While social desirability and other response biases are possible in our self-report survey, reliable and valid estimates of risky behaviors is possible with surveys [41], including with COVID-19 [18]. Moreover, relatively economical, rapid turn-around national household surveys such as AmeriSpeak can be complemented with more extensive and costly efforts to achieve more granular understanding of individual behaviors and survey responses, as well as to investigate the circumstances, behaviors, and beliefs of severely-vulnerable Americans and others who are not effectively reached through surveys.

## Conclusion

Our data suggest that most American adults have adopted at least some protective behaviors to reduce the risk of COVID-19 transmission and infection. We also find uneven take-up of such measures. Younger adults, low-income respondents, those with CJI histories, and those identifying themselves as Republican were less likely to report protective behaviors. These findings are important for prevention. They will remain highly relevant as vaccines become available, given the complementarities between vaccines and protective behaviors, as well as the many challenges in delivering vaccines.

We found little relationship between the emergence of local hotspots or state public health policies with respondents’ protective behaviors. Low prevalence of protective behaviors may have accelerated local disease spread. One or both of these factors may have necessitated more aggressive state measures. Alternatively, respondents’ protective behaviors may not be very responsive to local public policies and epidemiological conditions.

At this writing, millions of Americans are starting to receive COVID-19 vaccines. However, vaccines alone may be insufficient to halt the current pandemic, at least in the near term [42], which means that we will need to continue to further understand barriers to prevention behaviors (e.g., masking and distancing) and testing associated with reducing the spread of COVID-19. More specifically, the nation will still require an effective COVID-19 prevention toolkit to slow disease spread. This toolkit must include existing recommended protective behaviors, COVID-19 testing, renewed contact tracing efforts designed in light of previous efforts, alongside evidence-based measures to promote vaccine uptake. Public health messaging must be designed and focused to achieve cultural competence across a diverse range of communities and populations that differ in their sociodemographic make-up, cultural and political identities. The public health community faces a key challenge to identify culturally competent messaging, delivered through trusted messengers, to promote and reinforce both vaccination and evidence-based protective behaviors.

Throughout, nationally representative surveys can help accomplish these tasks. Such research can help monitor how protective behaviors and vaccine hesitance vary across the US, particularly among those groups at greatest risk. It can also assist in the development of focused epidemiological surveillance, and the testing of public health messaging and other COVID-19 prevention interventions.

## Supporting information

S1 Appendix AmeriSpeak survey questions. (DOCX)

S1 Data (CSV)

S2 Data (XLSX)

## References

[1] Coronavirus Resource Center: Johns Hopkins University 2020 [Available from: https://coronavirus.jhu.edu/map.html.

[2] Webb Hooper M, Nápoles AM, Pérez-Stable EJ. COVID-19 and Racial/Ethnic Disparities. Jama. 2020. doi: 10.1001/jama.2020.8598 32391864 PMC9310097

[3] Kang SJ, Jung SI. Age-Related Morbidity and Mortality among Patients with COVID-19. Infect Chemother. 2020;52(2):154–64. doi: 10.3947/ic.2020.52.2.154 32537961 PMC7335648

[4] Holuka C, Merz MP, Fernandes SB, Charalambous EG, Seal SV, Grova N, et al. The COVID-19 Pandemic: Does Our Early Life Environment, Life Trajectory and Socioeconomic Status Determine Disease Susceptibility and Severity? Int J Mol Sci. 2020;21(14). doi: 10.3390/ijms21145094 32707661 PMC7404093

[5] Jenkins WD, Bolinski R, Bresett J, Van Ham B, Fletcher S, Walters S, et al. COVID-19 During the Opioid Epidemic—Exacerbation of Stigma and Vulnerabilities. J Rural Health. 2020. doi: 10.1111/jrh.12442 32277731 PMC7262104

[6] Nafilyan V, Islam N, Ayoubkhani D, Gilles C, Katikireddi SV, Mathur R, et al. Ethnicity, Household Composition and COVID-19 Mortality: A National Linked Data Study. medRxiv. 2020:2020.11.27.20238147. 10.1177/0141076821999973 PMC7994923 33759630

[7] Volkow ND. Collision of the COVID-19 and Addiction Epidemics. Ann Intern Med. 2020;173(1):61–2. doi: 10.7326/M20-1212 32240293 PMC7138334

[8] Wang QQ, Kaelber DC, Xu R, Volkow ND. COVID-19 risk and outcomes in patients with substance use disorders: analyses from electronic health records in the United States. Mol Psychiatry. 2020:1–10. 10.1038/s41380-020-00880-7 PMC7488216 32929211

[9] Novel Coronavirus (2019-nCoV) Advice for the Public: World Health Organization; 2020b [updated n.d. Available from: https://www.who.int/emergencies/diseases/novel-coronavirus-2019/advice-for-public.

[10] 2019 Novel Coronavirus: Centers for Disease Control and Prevention; 2020a [Available from: https://www.cdc.gov/coronavirus/2019-ncov/about/transmission.html.

[11] Pradhan D, Biswasroy P, Kumar Naik P, Ghosh G, Rath G. A Review of Current Interventions for COVID-19 Prevention. Arch Med Res. 2020;51(5):363–74. doi: 10.1016/j.arcmed.2020.04.020 32409144 PMC7190516

[12] Coronavirus Disease 2019: How to protect yourself & others.: Centers for Disease Control and Prevention; 2020b [Available from: https://www.cdc.gov/coronavirus/2019-ncov/prevent-getting-sick/prevention.html.

[13] Clements JM. Knowledge and behaviors toward COVID-19 among US residents during the early days of the pandemic: cross-sectional online questionnaire. JMIR public health and surveillance. 2020;6(2):e19161. doi: 10.2196/19161 32369759 PMC7212816

[14] NORC. Technical Overview of the AmeriSpeak Panel: NORC’s Probability-Based Household Panel. Chicago, IL: NORC; 2020.

[15] Bilgen I, Dennis JM, Ganesh N. Nonresponse Follow-up Impact on AmeriSpeak Panel Sample Composition and Representativeness. Chicago, IL: NORC; 2018.

[16] Montgomery R, Dennis JM, Ganesh N. Response rate calculation methodology for recruitment of a two-phase probability-based panel: the case of AmeriSpeak. 2016.

[17] Dennis JM. AmeriSpeak Omnibus Field Report. NORC at the University of Chicago; 2019.

[18] Cawthon PM, Orwoll ES, Ensrud KE, Cauley JA, Kritchevsky SB, Cummings SR, et al. Assessing the Impact of the COVID-19 Pandemic and Accompanying Mitigation Efforts on Older Adults. J Gerontol A Biol Sci Med Sci. 2020;75(9):e123–e5. doi: 10.1093/gerona/glaa099 32307522 PMC7188163

[19] Anselin L. Local indicators of spatial association—LISA. Geographical analysis. 1995;27(2):93–115.

[20] Anselin L. A Local Indicator of Multivariate Spatial Association: Extending Geary’s c. Geographical Analysis. 2019;51(2):133–50.

[21] Anselin L, Li X. Operational local join count statistics for cluster detection. Journal of Geographical Systems. 2019;21(2):189–210. doi: 10.1007/s10109-019-00299-x 31171898 PMC6546301

[22] US Coronavirus Cases and Deaths: USA Facts; 2020 [Available from: https://usafacts.org/visualizations/coronavirus-covid-19-spread-map.

[23] Anselin L, Syabri I, Kho Y. GeoDa: An Introduction to Spatial Data Analysis. In: Fischer MM, Getis A, editors. Handbook of Applied Spatial Analysis: Software Tools, Methods and Applications. Berlin, Heidelberg: Springer Berlin Heidelberg; 2010. p. 73–89.

[24] Bethlehem J, Cobben F, Schouten B. The Use of Response Propensities. Handbook of Nonresponse in Household Surveys: John Wiley & Sons; 2011. p. 327–52.

[25] Guiahi M, Helbin PE, Teal SB, Stulberg D, Sheeder J. Patient Views on Religious Institutional Health Care. JAMA Netw Open. 2019;2(12):e1917008. doi: 10.1001/jamanetworkopen.2019.17008 31880794 PMC6991194

[26] McGinty EE, Presskreischer R, Han H, Barry CL. Psychological Distress and Loneliness Reported by US Adults in 2018 and April 2020. Jama. 2020;324(1):93–4. doi: 10.1001/jama.2020.9740 32492088 PMC7270868

[27] Mumford EA PJ, Villanti AC, Evans, WD. E-cigarette Beliefs: Testing a Relative Risk Message in a Representative US Sample. Tobacco Regulatory Science. 2019;5(2).

[28] Gamma A. Ebola Prevention Research: The Role of Threat in Ebola Prevention Behaviours. 2019.

[29] Van der Pligt J. Risk perception and self-protective behavior. European Psychologist. 1996;1(1):34–43.

[30] Hamel LS A. Is There a Widening Gender Gap in Coronavirus Stress?2020. Available from: https://www.kff.org/policy-watch/is-there-widening-gender-gap-in-coronavirus-stress/.

[31] Yıldırım M, Geçer E, Akgül Ö. The impacts of vulnerability, perceived risk, and fear on preventive behaviours against COVID-19. Psychol Health Med. 2020:1–9. 10.1080/13548506.2020.1776891 32490689

[32] Dubey MJ, Ghosh R, Chatterjee S, Biswas P, Chatterjee S, Dubey S. COVID-19 and addiction. Diabetes Metab Syndr. 2020;14(5):817–23. doi: 10.1016/j.dsx.2020.06.008 32540735 PMC7282772

[33] Butt C, Playne D, Hawick K, editors. Comparing Collective Behaviour of Socio-Physical Models. Proceedings of the International Conference on Modeling, Simulation and Visualization Methods (MSV); 2014: The Steering Committee of The World Congress in Computer Science, Computer.

[34] Litwin H, Levinsky M. Network-exposure severity and self-protective behaviors: The case of COVID-19. Innovation in Aging. 2021;5(2):igab015. doi: 10.1093/geroni/igab015 34131592 PMC8136077

[35] Iwamura Y, Tanimoto J. Realistic decision-making processes in a vaccination game. Physica A: Statistical Mechanics and its Applications. 2018;494:236–41.

[36] Kabir KA, Risa T, Tanimoto J. Prosocial behavior of wearing a mask during an epidemic: an evolutionary explanation. Scientific Reports. 2021;11(1):1–14. doi: 10.1038/s41598-020-79139-8 34135413 PMC8209058

[37] Macias Gil R, Marcelin JR, Zuniga-Blanco B, Marquez C, Mathew T, Piggott DA. COVID-19 Pandemic: Disparate Health Impact on the Hispanic/Latinx Population in the United States. J Infect Dis. 2020;222(10):1592–5. doi: 10.1093/infdis/jiaa474 32729903 PMC7454709

[38] Yancy CW. COVID-19 and African Americans. Jama. 2020;323(19):1891–2. doi: 10.1001/jama.2020.6548 32293639

[39] Leininger LP H. We’re public health experts. We need to do a better job of talking to conservatives. The Washington Post. 2020, October 12.

[40] Schneider J. Community network driven COVID- COVID-19 testing of vulnerable populations in the Central United States. Grant Number: UG1DA050066, Sept. 30, 2020—August 31, 2021.: National Institutes of Health; 2020.

[41] Thornberry TP, Krohn M. The Self-Report Method for Measuring Delinquency and Crime. Criminal Justice. Measurement and Analysis of Crime and Justice. 42000. p. 33–83.

[42] Paltiel AD, Schwartz JL, Zheng A, Walensky RP. Clinical outcomes of a COVID-19 vaccine: implementation over efficacy. Health Affairs. 2020;40:42–52. doi: 10.1377/hlthaff.2020.02054 33211536 PMC7931245

