# Supplementary material for: National variability in Americans’ COVID-19 protective behaviors: Implications for vaccine roll-out
Source: PLoS One. 2021 Nov 5;16(11):e0259257. doi: 10.1371/journal.pone.0259257 (PMC8570526; doi:10.1371/journal.pone.0259257)
Supplement: S1 Appendix — (DOCX) [file pone.0259257.s001.docx]

# **AmeriSpeak Survey Questions**

1. Have you been tested for COVID-19 (coronavirus)? If so, what was the result?
   1. I have been tested and I tested positive (I had coronavirus)
   2. I have been tested and I tested negative (I did not have coronavirus)
   3. I have been tested and I do not know the result
   4. I had COVID-19 symptoms (e.g., fever, body aches, upper respiratory distress/shortness of breath, temporary loss of smell, cough, diarrhea or vomiting), but did not get tested
   5. I have not been tested
2. Which of the following have you done in reaction as a general response to the coronavirus pandemic?

| **Response options** | **1 = Yes** | **2 = No** |
| --- | --- | --- |
| a. Cancelled a doctor’s (or other healthcare) appointment | 1 | 2 |
| b. Prayed | 1 | 2 |
| c. Had a “telehealth visit” with a doctor or other health care provider (such as over the phone or on a video chat) | 1 | 2 |
| d. Avoided in-person contact with friends or family | 1 | 2 |
| e. Washed/sanitized hands more than usual | 1 | 2 |
| f. Isolation from other person(s) who lives with you | 1 | 2 |
| g. Cancelled/postponed work or school activities | 1 | 2 |
| h. Cancelled/postponed travel | 1 | 2 |
| i. Have not left my home | 1 | 2 |
| j. I have done other things to keep myself safe | 1 | 2 |
| k. Working from home | 1 | 2 |
| l. Limiting interactions with others to groups of 10 or less | 1 | 2 |
| m. Keeping a 6-foot radius when interacting with people you do not live with | 1 | 2 |
| n. Stocking up on extra food | 1 | 2 |
| o. Stocking up on extra cleaning supplies | 1 | 2 |
| p. Wearing a mask when leaving home | 1 | 2 |
| q. I have done other things to keep myself safe | 1 | 2 |
| r. I am not taking any of these steps | 1 | 2 |
| s. None of the above | 1 | 2 |

1. In your lifetime, have you ever misused opioids/prescription pain medication (used in a way other than prescribed or developed a problem with them)?
   1. No
   2. Yes
2. Have you ever been convicted of any misdemeanor or felony crime?
   1. Yes, most recently within the past year
   2. Yes, most recently over a year ago
   3. No
3. Have you ever been incarcerated in jail or prison?
   1. Yes, most recently within the past year
   2. Yes, most recently over a year ago
   3. No

**AmeriSpeak provides the following demographic variables**

1. Respondent gender
   1. Unknown
   2. Male
   3. Female
2. Age: [number entry]
3. Combined race/ethnicity
   1. White, non-Hispanic
   2. Black, non-Hispanic
   3. Other, non-Hispanic
   4. Hispanic
   5. 2+, non-Hispanic
   6. Asian, non-Hispanic
4. Education
   1. No high school diploma
   2. High school graduate or equivalent
   3. Some college
   4. BA or above
5. Current Employment Status
   1. Working - as a paid employee
   2. Working - self-employed
   3. Not working - on temporary layoff from a job
   4. Not working - looking for work
   5. Not working - retired
   6. Not working - disabled
   7. Not working – other
6. Household Income
   1. Less than $5,000
   2. $5,000 to $9,999
   3. $10,000 to $14,999
   4. $15,000 to $19,999
   5. $20,000 to $24,999
   6. $25,000 to $29,999
   7. $30,000 to $39,999
   8. $40,000 to $49,999
   9. $50,000 to $59,999
   10. $60,000 to $74,999
   11. $75,000 to $84,999
   12. $85,000 to $99,999
   13. $100,000 to $124,999
   14. $125,000 to $149,999
   15. $150,000 to $174,999
   16. $175,000 to $199,999
   17. $200,000 or more
7. Region
   1. Northeast
   2. Midwest
   3. South
   4. West
8. Metropolitan Area Flag
   1. Non-metro area
   2. Metro area
9. Political Party Affiliation
   1. Democrat
   2. Lean Democrat
   3. Neither/Don’t Lean/Independent
   4. Lean Republican
   5. Republican
